# Differential Proteomic Analysis Reveals the Effect of Calcium on *Malus baccata* Borkh. Leaves under Temperature Stress

**DOI:** 10.3390/ijms18081755

**Published:** 2017-08-11

**Authors:** Lijie Li, Hong Su, Huaiyu Ma, Deguo Lyu

**Affiliations:** 1 College of Horticulture, Shenyang Agricultural University, Shenyang 110866, China; erjie13@syau.edu.cn; 2 Tianjin Facility Agriculture Research Institute, Tianjin 301700, China; suhong19871014@126.com; 3 Key Lab of Fruit Quality Development and Regulation of Liaoning province, Shenyang 110866, China

**Keywords:** temperature stress, calcium, iTRAQ, leaves, *M. baccata*

## Abstract

In the cool apple-producing areas of northern China, air temperature during early spring changes in a rapid and dramatic manner, which affects the growth and development of apple trees at the early stage of the growing season. Previous studies have shown that the treatment of calcium can increase the cold tolerance of *Malus baccata* Borkh., a widely-used rootstock apple tree in northern China. To better understand the physiological function of calcium in the response of *M. baccata* to temperature stress, we analyzed the effect of calcium treatment (2% CaCl_2_) on *M. baccata* leaves under temperature stress. Physiological analysis showed that temperature stress aggravated membrane lipid peroxidation, reduced chlorophyll content and induced photo-inhibition in leaves, whereas these indicators of stress injuries were alleviated by the application of calcium. An isobaric tags for relative and absolute quantitation (iTRAQ)-based proteomics approach was used in this study. Among the 2114 proteins that were detected in *M. baccata* leaves, 41, 25, and 34 proteins were differentially regulated by the increasing, decreasing, and changing temperature treatments, respectively. Calcium treatment induced 9 and 15 proteins after increasing and decreasing temperature, respectively, in comparison with non-treated plants. These calcium-responsive proteins were mainly related to catalytic activity, binding, and structural molecule activity. Hierarchical cluster analysis indicated that the changes in abundance of the proteins under increasing temperature and changing temperature treatments were similar, and the changes in protein abundance under decreasing temperature and increasing temperature with calcium treatment were similar. The findings of this study will allow a better understanding of the mechanisms underlying the role of calcium in *M. baccata* leaves under temperature stress.

## 1. Introduction

In the cool apple-producing areas of northern China, the air temperature during early spring fluctuates in a rapid and drastic manner, which is harmful to the formation of buds and leaves, and severely impedes the growth and development of apple trees [1]. Previous studies have investigated some physiological-biochemical characteristics of *Malus baccata* roots under temperature stress, and have shown that changing temperature decreases root vitality and has a negative effect on mitochondrial function and nitrogen and respiratory metabolism [1,2]. The changing temperature stress can also damage the photosynthetic function and affect the anti-oxidation activities of leaves in *M. baccata* [3]. Thus, it is important to improve the tolerance of apple trees to the stress caused by rapid changes in temperature.

Photosynthesis determines the yield of crops. It is one of the most obvious physiological processes affected by temperature stress. Photosynthetic pigments, photosystems, electron transfer system, and carbon dioxide reduction pathways are important parts of photosynthesis; damage to any of these parts can inhibit the photosynthesis of plants [4]. Chlorophyll fluorescence has often been used as a probe for plant photosynthesis to determine the effect of environmental stresses on the photosystem (PS) II [5]. Temperature stress can reduce chlorophyll synthesis [6]. Dutta et al. reported that high temperature stress can inhibit chlorophyll synthesis [7].

Calcium plays a critical role in plant stress responses by regulating physiological and biochemical processes. Cold stress often leads to an increase in free Ca^2+^ in plants, which is followed by cold-induced protein phosphorylation and the accumulation of the cold acclimation-specific genes that help in the adaptation of plants to cold stress [8,9]. Calcium can bind to the proteins and lipids at membrane surfaces, affects the pH of cells, and inhibits solute leakage from the cytoplasm, thereby contributing to the maintenance of cell membranes [10]. Moreover, calcium can regulate the swelling and expansion of guard cells in the leaves and stomatal aperture [11]. Several studies have indicated that calcium acts as a regulator of plants to respond against environmental stresses such as heat, cold, drought and salt [12,13,14,15]. Different studies have shown that the exogenous application of calcium can alleviate the damage to plant membranes and the lipid peroxidation caused by stress, increase the activity of antioxidant enzymes and the content of chlorophyll, effectively improve plant photosynthesis, and can significantly enhance the resistance of plants to stress [15,16]. In addition, as an intracellular second messenger, Ca^2+^ plays an important role in plant signal transduction under environmental stimuli (e.g., cold, drought, salt) [17,18,19,20]. Calcium improves plant cold resistance through two methods: one is the maintenance of the structure of the cell wall and cell membrane, and an improvement in the activity of protective enzymes; the other is the transfer of low-temperature signals and the induction of the expression of cold resistance genes [13]. However, the specific mechanism for the involvement of calcium in the response of *M. baccata* leaves to changing temperature stress remains unclear.

Differential proteomics can reflect the physiological state of plants and regulatory mechanisms that operate under different conditions. In general, proteins that are differentially abundant would be associated with plant stress responses. In recent years, studies on temperature stress using proteomics have become popular; these involve an exploration of the mechanism of adaptation of plants to temperature stress through the analysis and comparison of the composition, quantity, and modification of proteins under different temperature conditions [21,22,23]. The proteins and genes associated with a plant’s response to high-temperature stress are mainly heat shock proteins (HSPs), whereas those involved in the response to low-temperature stress are mostly chloroplast components, or play roles in reactive oxygen species (ROS) detoxification and energy production [23,24]. Two-dimensional electrophoresis (2-DE) is a traditional technique in comparative quantitative proteomic studies. However, owing to limitations of 2-DE, such as the detection of only a fraction of proteins, several other high-throughput techniques have been developed and used in different applications. The isobaric tags for relative and absolute quantitation (iTRAQ) labeling technique is one of the reliable techniques that allows the quantitative analysis of proteins starting from the peptide level. It has a large coverage on the protein abundance, and enables the identification and accurate quantitation of proteins from multiple samples [25]. The iTRAQ labeling technique has been used to explore the molecular mechanisms underlying the response of several plants to environmental stresses [26,27,28]. It has also been used in proteomics studies in determining the role of calcium in the response of plants to stress [29,30,31].

*M. baccata* is widely used as an apple rootstock because of its high cold tolerance. In this study, the seedlings of *M. baccata* were exposed to rapidly-changing temperature by simulating the characteristics of early-spring air temperature in the cool climatic region of northern China and to calcium treatment. iTRAQ was adopted to assess the changes in the proteome of *M. baccata* leaves under changing temperature stress and calcium treatment. In addition, we determined photosynthetic parameters in order to unravel the internal mechanism of the role of exogenously applied calcium in the response of *M. baccata* leaves to temperature stress.

## 2. Results

### 2.1. Changes in Cytomembrane Integrity and MDA Content

**Figure 1:**
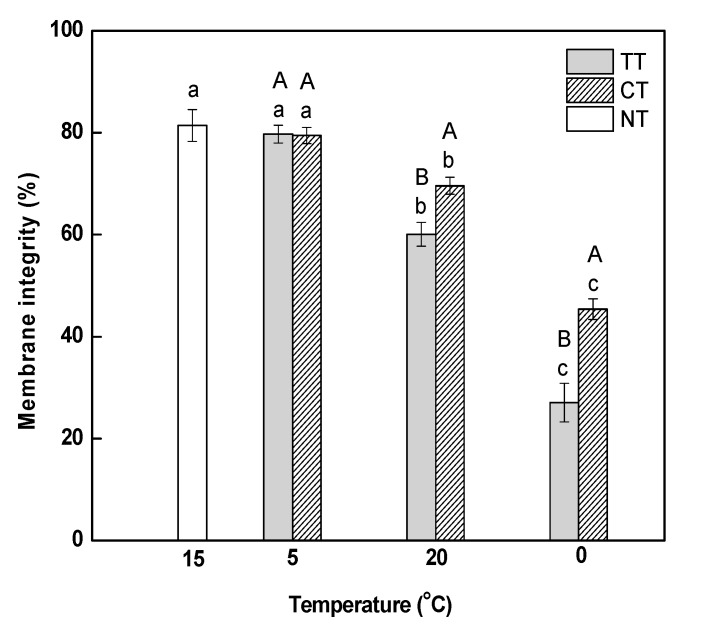
Effect of calcium on cytomembrane integrity in *M. baccata* leaves under temperature stress. **TT**, temperature treatment; **CT**, calcium treatment; **NT**, control. Lowercase letters above the bars indicate significant difference at *p* < 0.05 (Duncan’s test) of different treatments at the same temperature. Capital letters above the bars indicate significant difference at *p* < 0.05 (Duncan’s test) of the same treatment at different temperature.

Electrolyte leakage, which reflects the effect of abiotic stress on membrane integrity, was detected using a conductivity meter. We observed that a gradual decrease in temperature from 15 to 5 °C did not affect membrane integrity in *M. baccata* leaves (Figure 1). Electrolyte leakage decreased continuously as the temperature increased from 5 to 20 °C and then decreased to 0 °C, which demonstrated that rapidly changing temperature brought a negative influence on the integrity of membranes in the leaves. The treatment of calcium (CT) could relieve the stress induced by rapidly-changing temperature on membrane integrity, but did not change the decreasing trend of electrolyte leakage.

**Figure 2:**
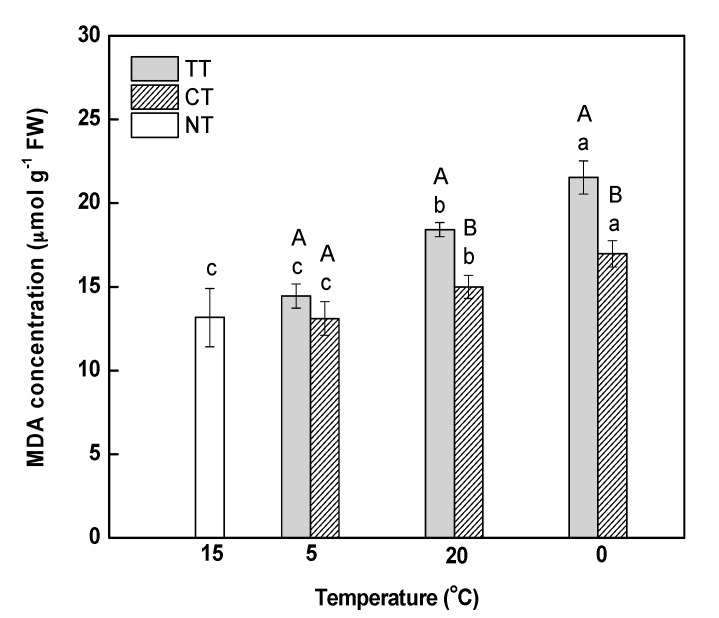
Effect of calcium on malondialdehyde (MDA) content in *M. baccata* leaves under temperature stress. **TT**, temperature treatment; **CT**, calcium treatment; **NT**, control. Lowercase letters above the bars indicate significant difference at *p* < 0.05 (Duncan’s test) of different treatments at the same temperature. Capital letters above the bars indicate significant difference at *p* < 0.05 (Duncan’s test) of the same treatment at different temperature.

The degree of oxidative damage in leaves was examined in terms of lipid peroxidation, as reflected by malondialdehyde (MDA) content. MDA content at 5 °C was slightly higher than that at 15 °C, which indicated that a gradual decrease in temperature from 15 to 5 °C caused no obvious injury to the cell membranes of the leaves (Figure 2). MDA content under temperature treatment (TT) increased significantly by 1.27 (at 20 °C) and 1.49-fold (at 0 °C) compared to the content at 5 °C. The treatment of calcium significantly decreased the MDA content in leaves under temperature stress; however, it was still significantly higher than that in the control (NT).

### 2.2. Changes in Chlorophyll Content

The effect of calcium on chlorophyll in *M. baccata* leaves under temperature stress is shown in Table 1. Total chlorophyll content in leaves decreased by 26.25% when the temperature gradually decreased from 15 to 5 °C. Total chlorophyll content changed markedly with the changes in temperature; it increased significantly after increasing temperature and decreased significantly after decreasing temperature. Total chlorophyll content at 0 °C was significantly lower than that at 5 °C. The treatment of calcium could increase total chlorophyll content to some extent but did not alter the changing trend during the temperature stress.

Chlorophyll *a* and chlorophyll *b* responded differently to the temperature stress. The changing trend in the content of chlorophyll *a* was similar to that of total chlorophyll, whereas the content of chlorophyll *b* at 5 °C was slightly higher than that at 15 °C and decreased continuously upon the imposition of changing-temperature stress. Calcium treatment had a positive influence on the accumulation of chlorophyll *a* under changing temperature, especially under low temperature (5 °C), but did not alter the trend of the change. Under calcium treatment, the content of chlorophyll *b* changed with the change in temperature. It decreased significantly at 5 °C compared to the content at the corresponding temperature in the absence of calcium, whereas calcium application enhanced the chlorophyll *b* content at 20 and 0 °C to some extent.

Chlorophyll *a*/*b* ratio remarkably decreased by 47.56% at 5 °C with respect to the ratio at 15 °C, and increased significantly by 1.92-fold after the temperature increased. The ratio decreased significantly by 14.3% after the temperature decreased. The treatment of calcium did not alter the varying tendency of chlorophyll *a*/*b* under temperature stress, but increased the ratio of chlorophyll *a*/*b* to some extent, and a significant increase was found at 5 °C (2.65-fold) compared to the corresponding condition without the application of calcium.

### 2.3. Analysis of Electron Transport Chain in Photosystem II (PSII)

**Figure 3:**
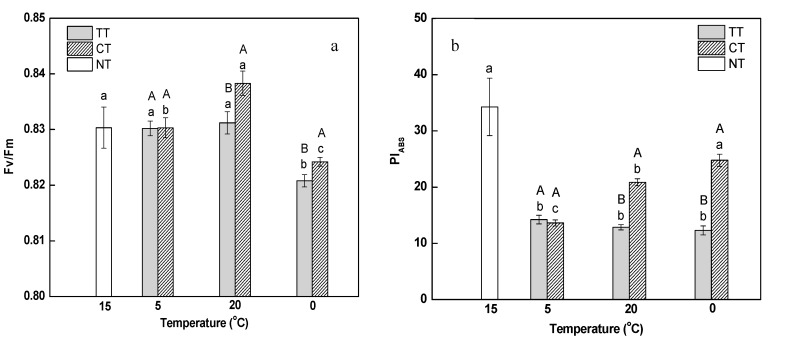
Effect of calcium on maximal photochemical efficiency of Photosystem II (Fv/Fm) (**a**) and performance index on absorption basis (PI_ABS_) (**b**) in *M. baccata* leaves under temperature stress. TT, temperature treatment; CT, calcium treatment; NT, control. Lowercase letters above the bars indicate significant difference at *p* < 0.05 (Duncan’s test) of different treatments at the same temperature. Capital letters above the bars indicate significant difference at *p* < 0.05 (Duncan’s test) of the same treatment at different temperature.

Maximal photochemical efficiency of photosystem II (PSII) (Fv/Fm) represents the potential quantum use efficiency of PSII. As shown in Figure 3a, both the gradual decrease in temperature from 15 to 5 °C as well as its rapid rise from 5 to 20 °C did not influence Fv/Fm in leaves; however, the ratio was remarkably reduced when the temperature dropped from 20 to 0 °C, indicating that PSII was inhibited during the decreasing of temperature. Fv/Fm did not vary in response to the treatment of calcium at 5 °C, but had a pronounced increase at 20 and 0 °C compared to the corresponding temperatures in the absence of calcium; this indicated that calcium can partially relieve the inhibition of PSII induced by temperature stress.

It has been reported that performance index on absorption basis (PI_ABS_) is more sensitive than Fv/Fm to some stresses, and can reflect the effect of stress on photosynthetic apparatus better [32]. PI_ABS_ decreased significantly and uniformly as the temperature changed from 15 to 5 °C (Figure 3b). It was also proved that PI_ABS_ was more sensitive to the low temperature than Fv/Fm. Under calcium treatment, PI_ABS_ increased continuously during the temperature stress, and was significantly higher than that under the temperature treatment at 20 °C (by 1.62-fold) and 0 °C (by 2.01-fold). It was indicated that calcium can effectively relieve the damage caused by changing temperature stress to photosynthetic apparatus.

### 2.4. Protein Identification and Relative Abundance Levels

**Figure 4:**
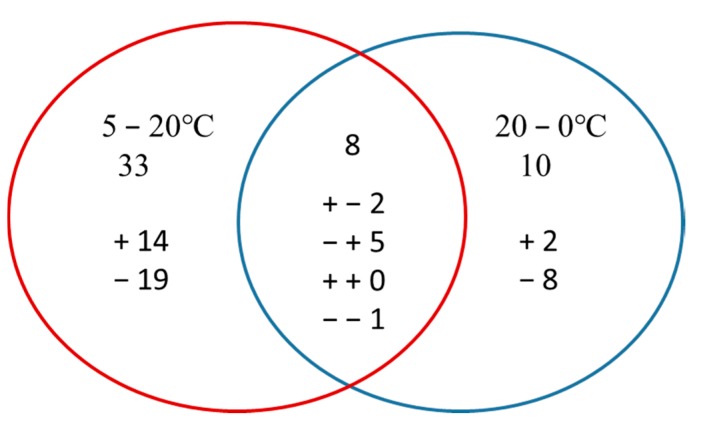
Venn diagram of differentially abundant proteins that were up or downregulated by an increase or decrease in temperature. The “+” and “−” indicate up and downregulated proteins, respectively. “+ − 2” means that, among the eight proteins, two proteins were upregulated in 5–20 °C while downregulated in 20–0 °C.

Overall, 2443 proteins with FDR < 1% were detected in this study (Tables S1 and S2). Only those proteins with a fold-change >1.2 (*p* < 0.05) were considered. Rapid increases and decreases in temperature affected the relative abundance of proteins in *M. baccata* leaves differently. Under conditions of rapid increase in temperature, 16 proteins were upregulated and 25 were downregulated, whereas seven were upregulated and 11 were downregulated under rapid decrease in temperature. The abundance of 34 proteins (10 up- and 24 downregulated) was significantly changed after the changing temperature treatment (Table 2), whereas that of eight proteins was significantly changed under conditions of both increasing and decreasing temperature. Among these eight proteins, one was downregulated under both the conditions, two were upregulated under increasing temperature and downregulated under decreasing temperature, and five were downregulated under increasing temperature and upregulated under decreasing temperature. Thirty-three and 10 proteins responded only to increasing and decreasing temperature, respectively (Figure 4).

Nine (3 up and 6 downregulated) proteins were significantly regulated under calcium treatment compared to the proteins expressed at 20 °C, and 15 proteins (8 up and 7 downregulated) were differentially accumulated at 0 °C (Table 2).

### 2.5. Molecular Function Analysis

**Figure 5:**
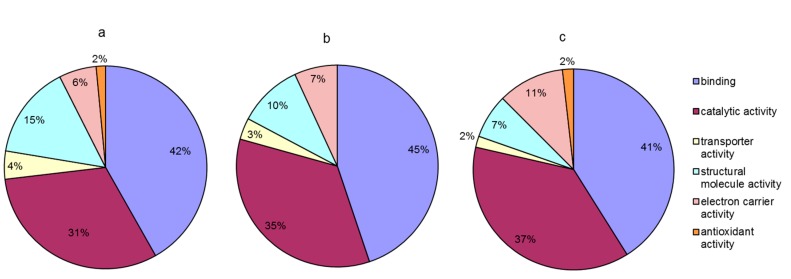
Molecular function of differentially abundant proteins under temperature stress. (**a**) Proteins responding to rapid increase in temperature (5–20 °C); (**b**) proteins responding to rapid decrease in temperature (20–0 °C); (**c**) proteins responding to the entire temperature treatment (5–20–0 °C).

Among the 117 differentially abundant proteins, 56 were characterized as hypothetical proteins without a specific function in the database. To obtain functional information about the proteins, we used the BLAST search for homologous proteins against the NCBI non-redundant protein database [33]. The e-value threshold was set to less than 1 × 10^−5^, and the best hit for each query sequence was taken account for gene ontology (GO) term matching. All differentially abundant proteins were classified according to their molecular functions on the basis of gene ontology (GO) annotation [34]. The proteins that responded to a rapid increase in temperature were assigned to six functional classes: binding (42%), catalytic activity (31%), structural molecule activity (15%), electron carrier activity (6%), transporter activity (4%), and antioxidant activity (2%) (Figure 5a). Proteins responding to rapidly decreasing temperature were assigned to five functional classes: binding (45%), catalytic activity (35%), structural molecule activity (10%), electron carrier activity (7%), and transporter activity (3%) (Figure 5b). Proteins responding to the entire temperature treatment were mainly involved in binding (41%), catalytic activity (37%), electron carrier activity (11%), structural molecule activity (7%), transporter activity (2%), and antioxidant activity (2%) (Figure 5c). Proteins in the categories of binding and catalytic activity were the most responsive to the stress caused by rapid changes in temperature. However, compared to that under rapidly increasing temperature, no proteins related to antioxidant activity were found differentially expressed under decreasing temperature. After the changing temperature treatment, the number of proteins related to electron carrier activity was increased and the number of proteins that serve as structural molecules was decreased.

**Figure 6:**
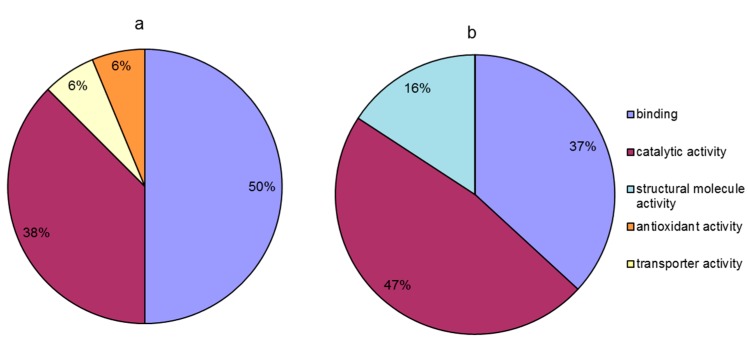
Molecular function of calcium-responsive proteins under temperature stress. (**a**) Calcium responsive proteins at 20 °C (20 °C + Ca vs. 20 °C); (**b**) calcium responsive proteins at 0 °C (0 °C + Ca vs. 0 °C).

Compared to the temperature treatment at 20 °C, proteins that were differentially accumulated at 20 °C in the presence of calcium were classified into four groups based on their molecular function (Figure 6a); these groups included binding (50%), catalytic activity (38%), transporter activity (6%), and antioxidant activity (6%). Moreover, compared to the temperature treatment at 0 °C, in the calcium treatment at 0 °C, the differentially abundant proteins were classified into three groups based on their molecular function (Figure 6b); these groups included catalytic activity (47%), binding (37%), and structural molecule activity (16%). The results indicated that pretreatment with calcium alleviated the injury to leaves induced by temperature stress, mainly through the regulation of the relative abundance of proteins with binding, catalytic activity and structural molecule activity. The differential effect of calcium pretreatment between the different temperature points was that, compared with that in decreasing temperature stage, in the increasing temperature stage (at 20 °C), calcium was involved in regulating the proteins related to transporter and antioxidant activities except the same molecular functions (binding and catalytic activity). However, compared to the increasing temperature stage (at 20 °C), calcium treatment mediated the regulation of the relative abundance of proteins related to structural molecule activity except the same molecular functions (binding and catalytic activity) at 0 °C (Figure 6).

### 2.6. Hierarchical Cluster Analysis

**Figure 7:**
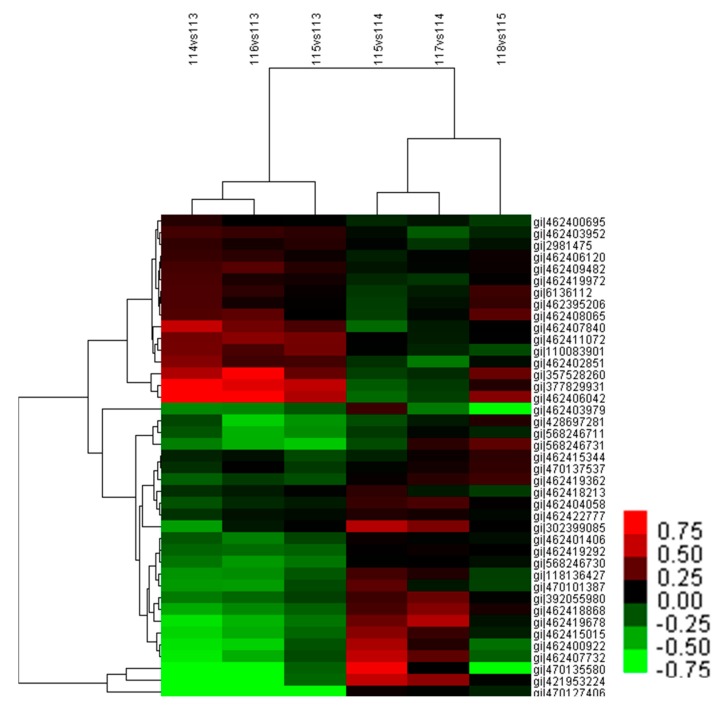
Hierarchical clustering analysis of differentially abundant proteins from *M. baccata* leaves under different treatments. The rows represent individual proteins. The proteins that increased and decreased in abundance are indicated in red and green, respectively. The proteins that were not changed are indicated in black. The intensity of the colors increased as the difference in abundance increased. 114 vs. 113: 5–20 °C; 116 vs. 113: 5 °C + Ca–5 °C; 115 vs. 113: 0–5 °C; 115 vs. 114: 20–0 °C; 117 vs. 114: 20 °C + Ca–20 °C; 118 vs. 115: 0 °C + Ca–0 °C.

Hierarchical cluster analysis was performed to further analyze the leaf proteome under temperature stress (Figure 7). Two main clusters were formed which contained 38 and three differentially abundant proteins, respectively. The 38 proteins in one cluster were classified into two sub-clusters: one sub-cluster included 16 proteins whose abundance mostly decreased under increasing temperature stage and under the changing temperature treatment, and increased under decreasing temperature stage. In addition, most of these proteins were upregulated by adding calcium at the increasing temperature stage; the other sub-cluster presented an opposite expression profile in each treatment. Moreover, the expression levels of the three proteins in the other cluster were similar to that in the second sub-cluster. The relative abundance patterns of the proteins under increasing temperature (114 vs. 113) and under the changing temperature stress (115 vs. 113) were similar, as was the case under decreasing temperature stress (115 vs. 114) and calcium treatment at 20 °C (117 vs. 114).

## 3. Discussion

It is well established that MDA content and electrolyte leakage are indicators of the level of injury to plant cell membranes [35]. In this study, electrolyte leakage was observed to decrease in *M. baccata* leaves after increasing temperature and decreasing temperature (Figure 1), which indicated that membrane integrity in the leaves was compromised. MDA content was increased under temperature stress, indicating that the temperature stress caused damage to the membranes and aggravated the peroxidation of membrane lipids. Previous studies have demonstrated that the application of calcium enhances the tolerance of plants to temperature stress by reducing cell membrane lipid peroxidation [12,36,37]. In this study, we found that calcium treatment alleviated the membrane damage induced by temperature stress.

Previous studies have also shown that low temperature decreased the content of photosynthetic pigments and Fv/Fm and induced photoinhibition in *Jatropha curcas* [38,39]. In the present study, rapid changes in temperature lead to the decrease in photosynthetic parameters, such as Fv/Fm, PI_ABS_, and in the content of photosynthetic pigment, which indicated that changing temperature treatment also resulted in damage to photosystem and caused photoinhibition in leaves. It has been reported that the treatment of calcium can enhance chlorophyll content, net photosynthetic rate, and carboxylation efficiency, resulting in the alleviation of temperature stress [12,13,16]. In a similar observation in this study, the addition of calcium to roots could alleviate the inhibition of photosynthesis induced by changing temperature. These results indicate that, under temperature stress, photoinhibition in leaves can be alleviated by enhancing the function of roots by adding calcium [1,2]. We also performed differential proteomic analysis of *M. baccata* leaves under conditions of rapid changes in temperature along with calcium treatment, the results of which are discussed below.

### 3.1. Binding Related Proteins

Heavy metal ATPase 1(HMA1) belongs to the heavy metal transporting P_1B_-type ATPase family, which is involved in delivering copper ions to the stroma, where they are essential for the detoxification of reactive oxygen species under adverse conditions [40]. Also, several heavy metal transporters such as HMA1 are implicated in Ca^2+^ transport [41]. In this experiment, the abundance of HMA1 was upregulated more than 8-fold after the treatment of calcium under conditions of rapid increase in temperature (Table 2). Studies have found that copper is an important redox cofactor involved in photosynthesis and electron transfer reactions [42]. Based on this result, in addition to the results of Fv/Fm and PI_ABS_, we hypothesize that the treatment of calcium protects *M. baccata* leaves from damages by up-regulating the abundance of HMA1 under increasing temperature conditions; this might promote the detoxification of ROS and might play a crucial role in maintenance of copper homeostasis.

The small GTP-binding proteins superfamily divided into five families; namely, Ras, Rho, Rab, Arf/ Sar and Ran. They are involved in a wide variety of cellular processes in eukaryotic cells, and some GTP-binding proteins might be involved in responding to abiotic stress [43,44]. The GTP-binding protein, secretion associated ras related GTPase 1A (SAR1A), encoded by SAR1A is essential for transporting endoplasmic reticulum cargo to the Golgi apparatus [45]. It has been reported that the AtSAR1 appeared to decline when tissue-culture cells are cold-shocked which adversely affect ER-to-Golgi transport of proteins [46]. We found that the abundance of SAR1A was up-regulated more than 1.7-fold in the treatment of calcium at 20 °C while was not changed during temperature stress (Table 2). This result suggests that changing temperature might not affect the membrane transport in *M. baccata* leaves. Because of the cross-talk between GTP-binding protein could be Ca^2+^ dependent [47], we suggested that calcium treatment could increase the membrane transport by increasing the abundance of SAR1A during the rapidly increasing temperature.

### 3.2. Catalytic Activity Related Proteins

Cinnamyl alcohol dehydrogenase (CAD) catalyzes the last step in the synthesis of the lignin precursors [48]. It has been reported that gene expression and activity of CAD are highly induced under cold stress [49,50,51]. After CaCl_2_ treatment, the activity of CAD in pears during cold storage was inhibited [52]. In line with previous studies, the abundance of CAD was increased in response to temperature stress in the present study (Table 2), indicating that increasing the lignin synthesis could be one of strategies adopted by *M. baccata* to respond to the temperature stress. However, CAD was downregulated under calcium treatment (Table 2). We suggest that the treatment of calcium decreased the abundance of CAD that was induced by temperature stress, which resulted in the level of synthesis of lignin being close to that in the control; this could also be a method for the alleviation of temperature stress.

Starch and sucrose are the main end-products of photosynthesis. In photosynthetic apparatus, UDP-glucose pyrophosphorylase (UGPase) primarily takes part in the synthesis of sucrose in plants, which provide UDPG for sucrose phosphate synthase (SPS) [53]. Wang et al. reported that cotton UGPase participates in sucrose/polysaccharide metabolism and the transcription level of *GhUGP* was increased in cotton under low temperature [54]. In this study, the abundance of UGPase was significantly upregulated at 20 °C (Table 2), which indicated that UGPase may accelerate the synthesis of sucrose during the rapidly increasing temperature. Granule-bound starch synthase (GBSS) is an enzyme that is responsible for amylose synthesis [55,56]. Sucrose phosphate phosphatase (SPP) is a key enzyme in sucrose synthesis that catalyzes the final step of this pathway [57]. In this study, GBSS1 was upregulated during the rapidly increasing temperature, which would have facilitated the synthesis of starch (Table 2). At 0 °C, calcium treatment significantly induced the abundance of GBSS1, whereas the abundance of SPP was obviously downregulated (Table 2). These results indicated that calcium treatment likely promoted the synthesis of starch, whereas it inhibited the synthesis of sucrose under temperature stress. It has been reported that changes of the cytosolic free-calcium concentration could regulate photosynthetic sucrose synthesis [58]. In addition, the decrease of sucrose synthesis allows more carbon to be stored as starch in the chloroplast [59]. So, the treatment of calcium in this study might promote the distribution of photosynthetic products to starch in *M. baccata* leaves. However, further experiments investigating the cytosolic free-calcium concentration will be needed to test this explanation. Coproporphyrinogen-III oxidase (CPO) is a vital enzyme in chlorophyll biosynthetic pathway [60]. It has been reported that CPO activity partially declined in cucumber seedlings under chilling stress [61]. With the change in chlorophyll content observed in this study (Table 1), the temperature stress might have inhibited the chlorophyll synthesis by decreasing the abundance of CPO (Table 2), and calcium treatment improved the chlorophyll content of *M. baccata* leaves by increasing the abundance of CPO under temperature stress (Table 2). Previous studies have found that the application of calcium inhibited the decrease in chlorophyll content under temperature stress, possibly by improving integrity of membrane or by alleviating the photo-oxidation [12,62]. Interestingly, in this experiment, calcium treatment significantly inhibited the decrease in membrane integrity and reduced the MDA concentration during the changing temperature stress (Figure 1 and Figure 2). Consequently, under temperature treatment, the treatment of calcium enhanced the photosynthetic function of leaves not only by improving membrane integrity, but also by promoting the abundance of CPO which improved the chlorophyll content.

Phosphoribulokinase (PRK) and Glyceraldehyde-3-phosphate dehydrogenase (GAPDH) are the key enzymes in Calvin cycle. PRK catalyzes the synthesis of ribose-1,5-phosphate from ribose-5-phosphate [63,64]. Ribulose-1,5-phosphate is utilized by ribulose-1,5-bisphosphate carboxylase/oxygenase for photosynthetic CO_2_ fixation in Calvin cycle [65]. Abrupt temperature reduction in winter wheat caused significant increase in relative abundance of PRK [66]. However, in *Arabidopsis thaliana*, PRK and GAPDH showed significant reductions in abundance during cold acclimation [67]. In this study, the down-regulation of PRK and GAPDH at the same time might have blocked CO_2_ assimilation under decreasing temperature (Table 2). In addition, PI_ABS_ was significantly decreased at 0 °C (Figure 3). Together with these results, we suggest that the decrease of PI_ABS_ in *M. baccata* leaves might have been due to the blockage of Calvin cycle when the temperature dropped rapidly.

Pantothenate kinase catalyzes the first step in the biosynthesis of coenzyme A (CoA), the precursor of acetyl coenzyme A, which is involved in the tricarboxylic acid (TCA) cycle [68]. In this study, the abundance of pantothenate kinase was significantly increased by calcium treatment at 0 °C compared to its abundance under temperature stress. The treatment of calcium might have facilitated the execution of TCA cycle by increasing the abundance of pantothenate kinase protein, and thereby improving the respiratory metabolism of leaves, which might have provided more energy and intermediate products for leaves in response to temperature stress. This could be another reason for the improved resistance of *M. baccata* leaves to rapidly changing temperature under the calcium treatment condition.

### 3.3. Transporter Activity Related Proteins

Plasma membrane H^+^-ATPase has a central function in the establishment and maintenance of ion equilibrium and in generating the proton gradient in the cytosol [69]. Vacuolar H^+^-PPase (VHP) is an electrogenic proton pump that generates the proton electrochemical gradient across the vacuolar membrane, which provides the motive force for the transport of ions and solutes, such as sugars, amino acids, and other compounds [70]. Both these phosphatases play key roles in the adaption of plants to a variety of abiotic stresses. Lee et al. demonstrated that the activity of plasma membrane H^+^-ATPase was inhibited by low-temperature stress, which had harmful effects on water transport of cucumber roots under temperature stress [71]. In addition, overexpression of *MdVHP1* could improve the tolerance of transgenic apple callus and tomato to low temperature stress [70]. In consonance with the results of previous studies, the abundance of plasma membrane H^+^-ATPase was significantly downregulated after the rapid increase in temperature with respect to that in the control (Table 2), and the abundance of VHP was significantly downregulated in both the increasing and decreasing temperature stages (Table 2). The down-regulation of plasma membrane H^+^-ATPase would, in turn, disturb the ion balance and that of VHP might inhibit the transport of solutes, which might have negative effects on *M. baccata* leaves under temperature stress.

### 3.4. Structural Molecule Activity Related Proteins

Ribosomal protein can regulate protein synthesis and cellular metabolism [72]. The induction of ribosomal genes might enhance the translation process or help proper ribosome functioning under low temperature [73]. However, in the present study, 40S ribosomal protein S6-like, 60S ribosomal protein L3-like, and 60S ribosomal protein L5-like were down-regulated under temperature stress (Table 2). Similarly, the relative abundance of 40S ribosomal protein S6-like and 60S ribosomal protein L3-like in plants after the treatment of calcium was also down-regulated (Table 2). Therefore, we suggest that the above mentioned ribosomal proteins were involved in the response of *M. baccata* leaves to temperature stress, but the effect of calcium on ribosomal proteins was not obvious.

### 3.5. Electron Carrier Activity Related Proteins

CP43 and CP47, encoded by *psbC* and *psbB*, respectively, are the core antenna complexes of PSII [74,75]. The function of these core antennas is to transfer excitation energy harvested by the outer antenna complexes (LHCII, CP29, CP26 and CP24) to the reaction center (RC) of PSII, where primary photochemistry occurs [76]. It has been reported that light-chilling downregulated thylakoid proteins by phosphorylation [77]; CP43 is one of the main thylakoid phosphoproteins. In the present study, the abundance of CP43 was downregulated at 20 and 0 °C, and that of CP47 was only downregulated at 20 °C (Table 2). The downregulation of proteins might due to phosphorylation. Moreover, Parida et al. reported that the abundance of CP47 declined by 30% under high salt stress compared to the abundance in the control; they suggested that this decrease might cause inefficient photon harvesting [78]. In our study, we found that PI_ABS_ was significantly decreased during the entire experiment (Figure 3). It is inferred that the decrease in CP43 and CP47 might inhibit the oxidation of water as well as the transfer of excitation energy from the peripheral antenna system to the RC of PSII, which might have ultimately caused the decline in PI_ABS_. From our result, we assume that the downregulation of cytochrome f might be another reason for the reduction of PI_ABS_ after the temperature stress (Table 2). Price et al. observed that there was a close correlation between the content of cytochrome f and photosynthetic rate [79]. Therefore, we suggest that rapid decrease in temperature influenced the electron transport chain of PSII by inhibiting the function of the acceptor portion, and the down-regulation of cytochrome f might play a role in inhibiting electron transport from PSII to PSI under rapid changes in temperature. D1 and D2 are the important components of PSII RC that play a crucial role in photosynthesis [80]. Low temperature inhibits the PSII repair cycle, degradation and de novo synthesis of the reaction centre D1 protein [81]. In the present study, the abundance levels of D1 and D2 proteins were significantly downregulated under the temperature stress (Table 2). It has been reported that net loss of functional PSII complexes was associated with net loss of D1 protein and D1 protein synthesis seems to play important role in sustaining PSII function [82]. The results in this study indicate that PSII function was damaged by temperature stress.

However, the proteins whose abundance was altered by calcium treatment, candidates to relieve the adverse effects of the changing temperature, may not be responsible for this in a direct way. The changes of the protein abundance may just be induced by calcium or due to an indirect effect of the calcium treatment. Therefore, the exact mechanism of these proteins needs further research.

## 4. Materials and Methods

### 4.1. Plant Material and Treatment

Seeds of *M. baccata* were stratified at 0–4 °C and were planted in 72-hole trays after germination and placed inside a greenhouse (25 ± 5 °C in day and 14 ± 3 °C in night). After 30 d, seedlings were transplanted into plastic pots (10 cm diameter and 10 cm height). Plantlets at 15-leaf stage were selected as the experimental materials and divided into two experimental groups: one group was watered with 100 mL distilled water and the other was treated with 100 mL of 2% (*w*/*v*) CaCl_2_. All plants were watered once before temperature treatment. Before their transfer to growth chamber (MLR-351H, SANYO, Osaka, Japan), the plantlets in the two groups were put in the dark for 16 h at 5 °C. The growth chamber was set at 5 °C (for 24 h) with a photosynthetic photon flux density of 150 μmol·m^−2^·s^−1^ (14 h); these plantlets were then exposed to the temperature treatment. The group treated with distilled water and not exposed to the temperature treatment was used as control (NT). The group of the control was harvested in a greenhouse (15 °C) at the time that the seedlings for temperature treatment were moved into the chamber. In the increasing temperature treatment, the temperature was increased from 5 to 20 °C (1 °C·h^−1^) and in the decreasing temperature treatment it was decreased from 20 to 0 °C (1 °C·h^−1^). The leaves were harvested at 5, 20 and 0 °C, respectively, after being kept at each temperature for 2 h. The group treated with distilled water under the above changing-temperature treatments (5 °C → 20 °C → 0 °C) was considered as temperature treatment (TT). The group treated with 2% (*w*/*v*) CaCl_2_ under the above changing temperature treatment was the calcium treatment (CT). Leaves from five seedlings were considered as one biological replicate and each treatment had three biological replicates.

### 4.2. Measurement of Chlorophyll a Fluorescence Parameters and Chlorophyll Content

The leaves were kept in the dark for 30 min before the measurement using a leaf clip. The chlorophyll *a* fluorescence transients were then measured using a plant efficiency analyzer-PEA (Hansatech Instruments Ltd, King’s Lynn, England). A saturating photon flux density of 3000 μmol·m^−2^·s^−1^ was set to induce the fluorescence of chlorophyll. The PEA automatically records fluorescence signals from 10 μs to 1 s. The parameters determined were as follows: φ_po_ (Fv/Fm) represents the maximum quantum yield of PSII (t = 0, φ_po_ = TRo/ABS); PI_ABS_ represents the overall activity of PSII, and was measured as follows: PI_ABS_ = (RC/ABS) × (φpo/(1 − φpo)) × (ψ0/(1 − ψ0)). Five replicate measurements were taken for each plant and, for each treatment at each target temperature, five plants were used for the measurement.

For determination of chlorophyll content, leaves (0.2 g) were homogenized in 80% acetone and then chlorophyll was estimated according to the method of Arnon [83]. Chlorophyll content was defined in terms of mg·g^−1^ FW.

### 4.3. Measurement of Membrane Integrity and MDA Concentration

Membrane integrity was measured according to the method of Satbhai and Naik [84]. Ten leaf discs (0.1 cm^−2^) were thoroughly rinsed three times with deionized water for 1 min each time, and immersed together in 10 mL deionized water at 40 °C for 30 min. Thereafter, the initial electrolyte leakage (E_1_) was recorded by a conductivity meter DDS-308A (Shanghai Precision Instruments Co. Ltd., Shanghai, China). Then, the solution was heated at 100 °C for 10 min before the final conductivity (E_2_) was determined. Membrane integrity was measured using the formula: Membrane integrity (%) = (1 − (E_1_/E_2_)) × 100.

MDA concentration was measured using a colorimetric assay [85]. Approximately 0.5 g of root tissue was homogenized in 10% trichloroacetic acid (0.25% 2-thiobarbituric acid). The reaction mixture was heated in a water bath (95 °C) for 30 min and then rapidly cooled in an ice bath. Thereafter, the mixture was centrifuged for 10 min (10,000× *g*, 4 °C). MDA content was determined by measuring the absorbance at 532 and 600 nm and the concentration was expressed as μmol·g^−1^ FW.

### 4.4. Protein Extraction from Leaves

Each sample (2 g) from three biological replicates in each treatment was weighed and ground to a powder with liquid nitrogen. The powder was dissolved in the lysis buffer I (8 M urea, 2 mM EDTA, 10 mM dithiothreitol (DTT), 150 mM NaCl, 1% TRITON X-100, 20 μL/mL protease inhibitor cocktail VI (Merck & Co., Kenilworth, NJ, USA), pH 8.0); the sample lysate was subjected to ultrasonic extraction at 200 W for 5 min and then was incubated in water bath at 36 °C for 1 h. The sample lysate was clarified by centrifugation at 15,000× *g* for 30 min at room temperature. Subsequently, the protein was added with cold 15% TCA, and transferred to −20 °C overnight. After centrifugation at 20,000× *g* for 15 min at 4 °C the precipitate was collected and washed with cold acetone for three times and air dried. The extracted proteins were resuspended in lysis buffer II (7 M urea, 2 M thiourea, 65 mM DTT, 4% 3-[(3-cholamidopropyl) dimethylammonio] propanesulfonate (CHAPS), 1 mM phenylmethylsulfonyl fluoride (PMSF), and 2 mM EDTA). A Bradford assay was used to measure the total protein concentrations. Each protein sample was digested and labeled for iTRAQ as described below.

### 4.5. Digestion and Isobaric Tags for Relative and Absolute Quantitation (iTRAQ) Labeling

Each protein sample (100 μg) was mixed sequentially with 10 μL digestion buffer (100 mM triethylammonium bicarbonate (TEAB), 0.05% *w*/*v* sodium dodecyl sulphate (SDS)) to a final concentration of 1 mg/mL, and was incubated in a water bath at 60 °C for 1 h. Subsequently, the sample was mixed with 1 μL of cysteine blocking reagent and kept at room temperature for 10 min. For iTRAQ experiment, 2 μg of trypsin was added to 100 μg of the protein solution for protein digestion and incubated at 37 °C for 12 h. iTRAQ labeling was done as per the protocols recommended by the manufacturer (AB Sciex, Framingham, MA, USA). Each tube of the labeling reagent was thawed and dissolved in 70 μL isopropanol. The control sample (harvested at 5 °C) was labelled with iTRAQ tag 113, the sample harvested at 20 °C was labelled with iTRAQ tag 114, the sample harvested at 0 °C was labelled with iTRAQ tag 115, the samples exposed to calcium treatment and harvested at 5 °C were labelled with iTRAQ tag 116, the samples exposed to calcium treatment and harvested at 20 °C were labelled with iTRAQ tag 117, and the samples exposed to calcium treatment and harvested at 0 °C were labelled with iTRAQ tag 118 (Figure S1). The labelled samples were kept at room temperature for 1 h, and then the six labelled samples were pooled together and centrifuged at 13000× *g* for 10 min. Finally, the supernatant was dried in a speedvac and then stored at −20 °C for further analysis.

### 4.6. Separation of the iTRAQ-Labelled Peptides by Reverse-Phase Chromatography

The pooled peptides were dissolved in 60 μL of buffer A (98% H_2_O and 2% ACN). The sample was then centrifuged at 13,000× *g* for 15 min and the supernatant was harvested. This step was repeated two times. Finally, 50 μL of the supernatant was used for reverse-phase chromatography on a RIGOL L-3000 system (Rigol, Beijing, China) equipped with an Agela Durashell C18(L) column (4.6 mm × 250 mm, 5 μm, 150A). The peptides were eluted at a flow rate of 0.7 mL/min with buffer B (98% ACN and 2% H_2_O). The absorbance at 214 nm was monitored. The fractions were collected every 90 s and then dried in a speedvac. Before mass spectrometry, all the fractions were redissolved in 0.1% FA for the next LC-MS/MS analysis.

### 4.7. Mass Spectrometric Identification of Proteins

Each fraction (2 μg) was loaded onto a precolumn (Acclaim PepMap 100 column, 2 cm × 100 μm, C18, 5 μm). Thereafter, the peptide mixture was eluted on a chromatographic column (EASY-Spray column, 12 cm × 75 μm, C18, 3 μm) with a flow rate of 350 nL/min and separated with a linear gradient of 1230% of mobile phase B(98% ACN and 0.1% FA) over 90 min. The reverse mobile phase A contained 98% H_2_O and 0.1% FA. The fractions were analyzed by MS using a 5600 TripleTOF analyzer (AB SCIEX, Foster City, CA, USA). The MS/MS scans in the *m*/*z* range from 350 to 1800 were recorded with 15 s dynamic exclusion setting.

### 4.8. Database Search and Data Analysis

The data were processed with ProteinPilot v.4.0 software (AB SCIEX, Foster City, CA, USA) using the Paragon and Progroup algorithm. The database of Rosaceae (74331 sequences) was downloaded from NCBI [86]. The parameters used for protein identification were as follows: digestion was with trypsin, static modification was carboxyamidomethylation, and dynamic modifications were protein *N*-terminal and oxidation (M). In the algorithm, the precursor ion mass tolerance and fragment ion mass tolerance were ±15 ppm and ±20 mmu, respectively. Two missed cleavages were set in this study. The false discovery rate (FDR) analysis of all the peptide and protein identifications was performed using the integrated tools in ProteinPilot (AB SCIEX). For data analysis, a 1.2-fold change in addition to a *p*-value < 0.05 was chosen for identification of significant differentially abundant proteins. The proteins with changes in the abundances >1.2-fold were considered to be differentially regulated. The classification of differentially abundant proteins was performed according to the gene ontology database.

### 4.9. Statistical Analysis

The statistical analysis was performed using SPSS 17.0 (SPSS Inc., Chicago, IL, USA). The multiple range test was performed at *p* = 0.05. All the values are shown as means ± standard error (SE).

## 5. Conclusions

**Figure 8:**
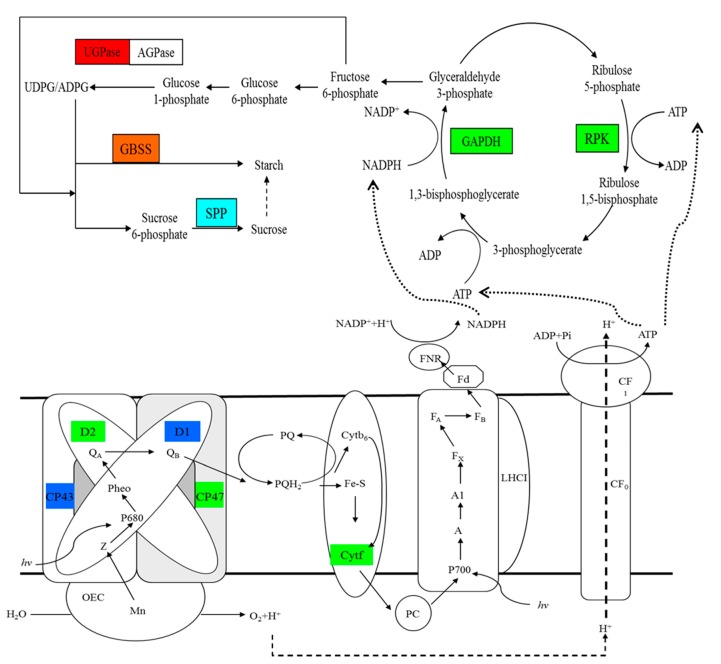
Changes in the photosynthetic electron transfer chain, Calvin cycle, and biosynthesis pathway of sucrose and starch in response to rapid changes in temperature in *M. baccata* leaves. The red box represents a protein only upregulated at 20 °C as compared to that at 5 °C. Green boxes indicate proteins only downregulated at 0 °C as compared to that at 5 °C. Oxford blue boxes indicate proteins downregulated at both 20 and 0 °C as compared to that at 5 °C. The light blue box indicates a protein only downregulated under calcium treatment at 0 °C. The orange box represents a protein upregulated at both 20 °C as compared to that at 5 °C, and under calcium treatment at 0 °C. AGPase, ADP-glucose pyrophosphorylase; Cytb6, cytochrome b6; Cytf, cytochrome f; FNR, ferrdoxin-NDAP + reductase; GAPDH, glyceraldehyde-3-phosphate dehydrogenase; GBSS, granule-bound starch synthase; LHCI, PSI light-harvesting complex; OEC, oxygen-evolving complex; PC, plastocyanin; Pheo, pheophytin; PQ, plastoquinone; PQH2, plastoquinol; PRK, phosphoribulokinase; and UGPase, UDP-glucose pyrophosphorylase.

Under temperature stress, the membrane integrity of cells in the leaves of *M. baccata* was destroyed, the membrane lipid peroxidation was increased, and the chlorophyll content was decreased, which resulted in the decline in photosynthetic function. The addition of calcium alleviated these adverse effects to a certain extent. Using the iTRAQ technique, we compared the proteins in *M. baccata* leaves under changing temperature stress and calcium treatment, and a total of 117 differentially abundant proteins were detected. These proteins could be divided into six categories according to their molecular functions, including binding, catalytic activity, structural molecule activity, electron carrier activity, transporter activity and antioxidant activity. The results showed that calcium participated in the response of *M. baccata* leaves to temperature stress mainly through the pathways related to light and the electron transfer chain, Calvin cycle, and the synthesis of starch and sucrose (Figure 8). The changing temperature stress leads to the negative regulation of the proteins related to photosynthesis (CPO, CP43, CP47, D1, D2, cytochrome f, and phosphoribulose kinase) in *M. baccata* leaves. The treatment of calcium improved the adaptability of leaves to temperature stress by upregulating the abundance of proteins such as GTP binding protein, pantothenate kinase, GBSS, etc. This study therefore provides further insights into the role of calcium in the response of *M. baccata* leaves to changing temperature stress.

## Figures and Tables

**Table 1:** Effect of calcium on the contents of pigments in *M. baccata* leaves under temperature stress.

Temperature	Treatment	Chlorophyll *a* (mg·g^−1^ FW)	Chlorophyll *b* (mg·g^−1^ FW)	Total Chlorophyll (mg·g^−1^ FW)	Chlorophyll *a*/*b* (mg·g^−1^ FW)
15 °C	NT	2.262 ± 0.074 a	0.746 ± 0.017 ab	3.009 ± 0.063 a	3.034 ± 0.158 a
5 °C	TT	1.359 ± 0.031 bB	0.860 ± 0.076 aA	2.219 ± 0.046 bA	1.591 ± 0.169 cB
	CT	1.814 ± 0.077 bA	0.440 ± 0.083 bB	2.253 ± 0.160 bA	4.215 ± 0.695 aA
20 °C	TT	2.113 ± 0.101 aA	0.690 ± 0.015 bA	2.803 ± 0.115 aA	3.060 ± 0.090 aA
	CT	2.196 ± 0.040 aA	0.716 ± 0.200 aA	2.912 ± 0.192 aA	3.232 ± 0.884 abA
0 °C	TT	1.162 ± 0.348 bA	0.438 ± 0.099 cA	1.600 ± 0.447 cA	2.622 ± 0.225 bA
	CT	1.412 ± 0.197 cA	0.503 ± 0.068 abA	1.915 ± 0.264 bA	2.806 ± 0.011 bA

Note: The data in Table 1 are means ± SE (*n* = 3). Lowercase letters indicate significant difference at *p* < 0.05 (Duncan’s test) of different treatments at the same temperature. Capital letters indicate significant difference at *p* < 0.05 (Duncan’s test) of the same treatment at different temperature. TT, temperature treatment; CT, calcium treatment; NT, control; and FW, fresh weight.

**Table 2:** List of differentially abundant proteins in *M. baccata* leaves under different treatments.

Accession No. ^a^	Protein Name	Plant Species ^b^	Coverage (%) ^c^	Peptides (95%) ^d^	iTRAQ Ratio	Expression Pattern ^e^
20 °C vs 5 °C						
gi|462406120	hypothetical protein PRUPE_ppa001062mg	*Prunus persica*	40.28	53	1.47	↑
gi|462409482	hypothetical protein PRUPE_ppa002222mg	*Prunus persica*	45.78	47	1.58	↑
gi|462403952	hypothetical protein PRUPE_ppa003093mg	*Prunus persica*	48.76	38	1.54	↑
gi|462415344	hypothetical protein PRUPE_ppa000130mg	*Prunus persica*	19.75	33	0.74	↓
gi|462400695	hypothetical protein PRUPE_ppa004306mg	*Prunus persica*	50.10	43	1.32	↑
gi|392055980	plasma membrane H^+^-ATPase	*Malus baccata* var. *xiaojinensis*	27.88	22	0.40	↓
gi|462419972	hypothetical protein PRUPE_ppa005369mg	*Prunus persica*	53.23	29	1.63	↑
gi|428697281	photosystem II CP43 chlorophyll apoprotein	*Fragaria virginiana*	34.88	39	0.59	↓
gi|462411072	hypothetical protein PRUPE_ppa000855mg	*Prunus persica*	29.05	26	2.09	↑
gi|462403979	hypothetical protein PRUPE_ppa002220mg	*Prunus persica*	28.76	22	0.36	↓
gi|462418213	hypothetical protein PRUPE_ppa000841mg	*Prunus persica*	15.04	14	0.69	↓
gi|6136112	UTP—glucose-1-phosphate uridylyltransferase;	*Pyrus pyrifolia*	37.79	14	1.66	↑
gi|462395206	hypothetical protein PRUPE_ppa004819mg	*Prunus persica*	36.73	15	1.64	↑
gi|568246711	photosystem II protein D1	*Pyrus spinosa*	40.23	21	0.53	↓
gi|462407840	hypothetical protein PRUPE_ppa010884mg	*Prunus persica*	40.95	28	3.50	↑
gi|568246731	photosystem I P700 apoprotein A1	*Pyrus spinosa*	16.40	14	0.39	↓
gi|110083901	dehydroascorbate reductase	*Malus domestica*	53.99	14	2.13	↑
gi|2981475	putative cinnamyl alcohol dehydrogenase	*Malus domestica*	34.46	11	1.41	↑
gi|462404058	hypothetical protein PRUPE_ppa000235mg	*Prunus persica*	7.75	10	0.54	↓
gi|462401406	hypothetical protein PRUPE_ppa010331mg	*Prunus persica*	43.87	18	0.52	↓
gi|462402851	hypothetical protein PRUPE_ppa002111mg	*Prunus persica*	23.22	13	2.33	↑
gi|462419292	hypothetical protein PRUPE_ppa009763mg	*Prunus persica*	27.24	13	0.47	↓
gi|568246730	photosystem I P700 apoprotein A2	Pyrus spinosa	18.53	15	0.42	↓
gi|302399085	TCP domain class transcription factor	*Malus domestica*	16.67	7	0.31	↓
gi|462419362	hypothetical protein PRUPE_ppa004904mg	*Prunus persica*	18.52	7	0.49	↓
gi|462408065	hypothetical protein PRUPE_ppa012896mg	*Prunus persica*	47.33	10	1.71	↑
gi|118136427	vacuolar H^+^-PPase	*Malus domestica*	6.85	4	0.33	↓
gi|357528260	granule-bound starch synthase GBSS1	*Malus domestica*	28.34	19	3.02	↑
gi|470135580	PREDICTED: 60S ribosomal protein L3-like	*Fragaria vesca* subsp. *vesca*	13.11	6	0.10	↓
gi|470137537	PREDICTED: coproporphyrinogen-III oxidase, chloroplastic-like	*Fragaria vesca* subsp. *vesca*	9.07	3	0.69	↓
gi|462419678	hypothetical protein PRUPE_ppa004988mg	*Prunus persica*	7.05	3	0.19	↓
gi|421953224	BIS1 biphenyl synthase	*Malus domestica*	11.79	5	0.12	↓
gi|377829931	rps7 gene product (chloroplast)	*Pentactina rupicola*	23.23	5	6.31	↑
gi|462400922	hypothetical protein PRUPE_ppa006524mg	*Prunus persica*	7.84	3	0.19	↓
gi|462415015	hypothetical protein PRUPE_ppa012878mg	*Prunus persica*	20.53	3	0.21	↓
gi|462422777	hypothetical protein PRUPE_ppa011659mg	*Prunus persica*	12.38	2	0.67	↓
gi|470101387	PREDICTED: 60S ribosomal protein L5-like	*Fragaria vesca* subsp. *vesca*	7.69	2	0.32	↓
gi|462406042	hypothetical protein PRUPE_ppa002386mg	*Prunus persica*	3.10	2	8.17	↑
gi|462407732	hypothetical protein PRUPE_ppa010094mg	*Prunus persica*	15.53	5	0.17	↓
gi|470127406	PREDICTED: 40S ribosomal protein S6-like	*Fragaria vesca* subsp. *vesca*	9.64	2	0.09	↓
gi|462418868	hypothetical protein PRUPE_ppa000009mg	*Prunus persica*	0.28	1	0.28	↓
0 °C vs 20 °C						
gi|462416702	hypothetical protein PRUPE_ppa001865mg	*Prunus persica*	52.93	68	0.48	↓
gi|462396213	hypothetical protein PRUPE_ppa025698mg	*Prunus persica*	34.58	31	0.65	↓
gi|462400695	hypothetical protein PRUPE_ppa004306mg	*Prunus persica*	50.10	43	0.73	↓
gi|568246744	cytochrome f	*Pyrus spinosa*	62.81	31	0.59	↓
gi|428697281	photosystem II CP43 chlorophyll apoprotein (chloroplast)	*Fragaria virginiana*	34.88	39	0.56	↓
gi|470128485	PREDICTED: phosphoribulokinase, chloroplastic-like	*Fragaria vesca* subsp. *vesca*	51.96	37	0.39	↓
gi|568246756	photosystem II CP47 chlorophyll apoprotein	*Pyrus spinosa*	29.72	32	0.65	↓
gi|462407840	hypothetical protein PRUPE_ppa010884mg	*Prunus persica*	40.95	28	0.45	↓
gi|381393060	glyceraldehyde-3-phosphate dehydrogenase A	*Pyrus* × *bretschneideri*	51.12	58	0.28	↓
gi|6177796	JPR ORF1	*Pyrus pyrifolia*	18.16	10	0.48	↓
gi|302399085	TCP domain class transcription factor	*Malus domestica*	16.67	7	3.13	↑
gi|470135580	PREDICTED: 60S ribosomal protein L3-like	*Fragaria vesca* subsp. *vesca*	13.11	6	4.79	↑
gi|462400607	hypothetical protein PRUPE_ppa002173mg	*Prunus persica*	9.49	6	0.58	↓
gi|421953224	BIS1 biphenyl synthase	*Malus domestica*	11.79	5	3.47	↑
gi|462400922	hypothetical protein PRUPE_ppa006524mg	*Prunus persica*	7.84	3	2.96	↑
gi|470101387	PREDICTED: 60S ribosomal protein L5-like	*Fragaria vesca* subsp. *vesca*	7.69	2	1.80	↑
gi|462405695	hypothetical protein PRUPE_ppa004869mg	*Prunus persica*	3.89	2	1.69	↑
gi|470103122	probable cadmium/zinc-transporting ATPase HMA1, chloroplastic-like	*Fragaria vesca* subsp. *vesca*	1.72	2	7.11	↑
0 °C vs 5 °C						
gi|462415344	hypothetical protein PRUPE_ppa000130mg	*Prunus persica*	19.75	33	0.58	↓
gi|568246744	cytochrome f	*Pyrus spinosa*	62.81	31	0.55	↓
gi|4165550	apgm	*Malus domestica*	47.76	27	1.43	↑
gi|428697281	photosystem II CP43 chlorophyll apoprotein (chloroplast)	*Fragaria virginiana*	34.88	39	0.33	↓
gi|462411072	hypothetical protein PRUPE_ppa000855mg	*Prunus persica*	29.05	26	2.11	↑
gi|462403979	hypothetical protein PRUPE_ppa002220mg	*Prunus persica*	28.76	22	0.52	↓
gi|470128485	PREDICTED: phosphoribulokinase, chloroplastic-like	*Fragaria vesca* subsp. *vesca*	51.96	37	0.54	↓
gi|462408129	hypothetical protein PRUPE_ppa006653mg	*Prunus persica*	59.35	30	0.59	↓
gi|568246756	photosystem II CP47 chlorophyll apoprotein	*Pyrus spinosa*	29.72	32	0.54	↓
gi|568246711	photosystem II protein D1	*Pyrus spinosa*	40.23	21	0.35	↓
gi|568246731	photosystem I P700 apoprotein A1	*Pyrus spinosa*	16.40	14	0.23	↓
gi|110083901	dehydroascorbate reductase	*Malus domestica*	53.99	14	2.09	↑
gi|381393060	glyceraldehyde-3-phosphate dehydrogenase A	*Pyrus* × *bretschneideri*	51.12	58	0.34	↓
gi|2981475	putative cinnamyl alcohol dehydrogenase	*Malus domestica*	34.46	11	1.32	↑
gi|462402866	hypothetical protein PRUPE_ppa000990mg	*Prunus persica*	12.03	11	0.43	↓
gi|462419292	hypothetical protein PRUPE_ppa009763mg	*Prunus persica*	27.24	13	0.48	↓
gi|568246730	photosystem I P700 apoprotein A2	*Pyrus spinosa*	18.53	15	0.41	↓
gi|462395764	hypothetical protein PRUPE_ppa008222mg	*Prunus persica*	35.88	12	0.69	↓
gi|568246726	photosystem II protein D2	*Pyrus spinosa*	24.93	23	0.22	↓
gi|313600351	beta-1,3-glucanase	*Malus hupehensis*	24.28	11	2.00	↑
gi|462422251	hypothetical protein PRUPE_ppa001168mg	*Prunus persica*	8.54	7	1.91	↑
gi|118136427	vacuolar H^+^-PPase	*Malus domestica*	6.85	4	0.52	↓
gi|462405098	hypothetical protein PRUPE_ppa008755mg	*Prunus persica*	14.69	8	0.41	↓
gi|470135580	PREDICTED: 60S ribosomal protein L3-like	*Fragaria vesca* subsp. *vesca*	13.11	6	0.49	↓
gi|470135491	PREDICTED: signal recognition particle 54 kDa protein, chloroplastic-like	*Fragaria vesca* subsp. *vesca*	11.78	6	2.91	↑
gi|470137537	PREDICTED: coproporphyrinogen-III oxidase, chloroplastic-like	*Fragaria vesca* subsp. *vesca*	9.07	3	0.64	↓
gi|462419678	hypothetical protein PRUPE_ppa004988mg	*Prunus persica*	7.05	3	0.42	↓
gi|421953224	BIS1 biphenyl synthase	*Malus domestica*	11.79	5	0.46	↓
gi|470101387	PREDICTED: 60S ribosomal protein L5-like	*Fragaria vesca* subsp. *vesca*	7.69	2	0.58	↓
gi|462406042	hypothetical protein PRUPE_ppa002386mg	*Prunus persica*	3.10	2	3.05	↑
gi|462407696	hypothetical protein PRUPE_ppa004527mg	*Prunus persica*	5.54	2	7.80	↑
gi|462405695	hypothetical protein PRUPE_ppa004869mg	*Prunus persica*	3.89	2	2.07	↑
gi|470127406	PREDICTED: 40S ribosomal protein S6-like	*Fragaria vesca* subsp. *vesca*	9.64	2	0.15	↓
gi|462424295	hypothetical protein PRUPE_ppa000786mg	*Prunus persica*	1.79	2	0.60	↓
20 °C + Ca vs 20 °C						
gi|462403952	hypothetical protein PRUPE_ppa003093mg	*Prunus persica*	48.76	38	0.51	↓
gi|462403979	hypothetical protein PRUPE_ppa002220mg	*Prunus persica*	28.76	22	0.41	↓
gi|2981475	putative cinnamyl alcohol dehydrogenase	*Malus domestica*	34.46	11	0.66	↓
gi|462402851	hypothetical protein PRUPE_ppa002111mg	*Prunus persica*	23.22	13	0.41	↓
gi|313600351	beta-1,3-glucanase	*Malus hupehensis*	24.28	11	0.39	↓
gi|114795078	glutathione S-transferase	*Pyrus communis*	24.51	5	0.60	↓
gi|421953224	BIS1 biphenyl synthase	*Malus domestica*	11.79	5	2.47	↑
gi|470129084	PREDICTED: GTP-binding protein SAR1A-like	*Fragaria vesca* subsp. *vesca*	11.40	3	1.79	↑
gi|470103122	probable cadmium/zinc-transporting ATPase HMA1, chloroplastic-like	*Fragaria vesca* subsp. *vesca*	1.72	2	8.09	↑
0 °C + Ca vs 0 °C						
gi|462403979	hypothetical protein PRUPE_ppa002220mg	*Prunus persica*	28.76	22	0.10	↓
gi|462418975	hypothetical protein PRUPE_ppa003053mg	*Prunus persica*	32.07	20	1.61	↑
gi|462405014	hypothetical protein PRUPE_ppa007588mg	*Prunus persica*	33.15	13	1.64	↑
gi|462402866	hypothetical protein PRUPE_ppa000990mg	*Prunus persica*	12.03	11	2.36	↑
gi|313600351	beta-1,3-glucanase	*Malus hupehensis*	24.28	11	0.39	↓
gi|462422598	hypothetical protein PRUPE_ppa001487mg	*Prunus persica*	16.32	12	0.56	↓
gi|462405098	hypothetical protein PRUPE_ppa008755mg	*Prunus persica*	14.69	8	2.38	↑
gi|357528260	granule-bound starch synthase GBSS1	*Malus domestica*	28.34	19	1.96	↑
gi|470135580	PREDICTED: 60S ribosomal protein L3-like	*Fragaria vesca* subsp. *vesca*	13.11	6	0.14	↓
gi|462409757	hypothetical protein PRUPE_ppa003798mg	*Prunus persica*	7.30	5	5.11	↑
gi|46093878	sucrose phosphate phosphatase	*Malus domestica*	13.18	5	0.76	↓
gi|462401293	hypothetical protein PRUPE_ppa009351mg	*Prunus persica*	19.59	5	0.21	↓
gi|470137537	PREDICTED: coproporphyrinogen-III oxidase, chloroplastic-like	*Fragaria vesca* subsp. *vesca*	9.07	3	1.41	↑
gi|470125921	PREDICTED: pantothenate kinase 2-like	*Fragaria vesca* subsp. *vesca*	6.72	6	1.38	↑
gi|470127406	PREDICTED: 40S ribosomal protein S6-like	*Fragaria vesca* subsp. *vesca*	9.64	2	0.75	↓

^a^ The protein ID from National Center for Biotechnology Information (NCBI); ^b^ The plant species that matched the peptides. Accession number was recorded as a reference for the identification in NCBI_Rosaceae database, and the species origin annotation of all matching proteins were belonging to the Rosaceae; ^c^ The amino acid sequence coverage for the identified proteins; ^d^ The number of distinct peptide sequences in the protein group; ^e^ ↑ upregulated, ↓ downregulated.

## Supplementary Materials

Supplementary materials can be found at www.mdpi.com/1422-0067/18/8/1755/s1.
