# Supplementary figures and images for: Differential Proteomic Analysis Reveals the Effect of Calcium on Malus baccata Borkh. Leaves under Temperature Stress
Source: Int J Mol Sci. 2017 Aug 11;18(8):1755. doi: 10.3390/ijms18081755 (PMC5578145; doi:10.3390/ijms18081755)

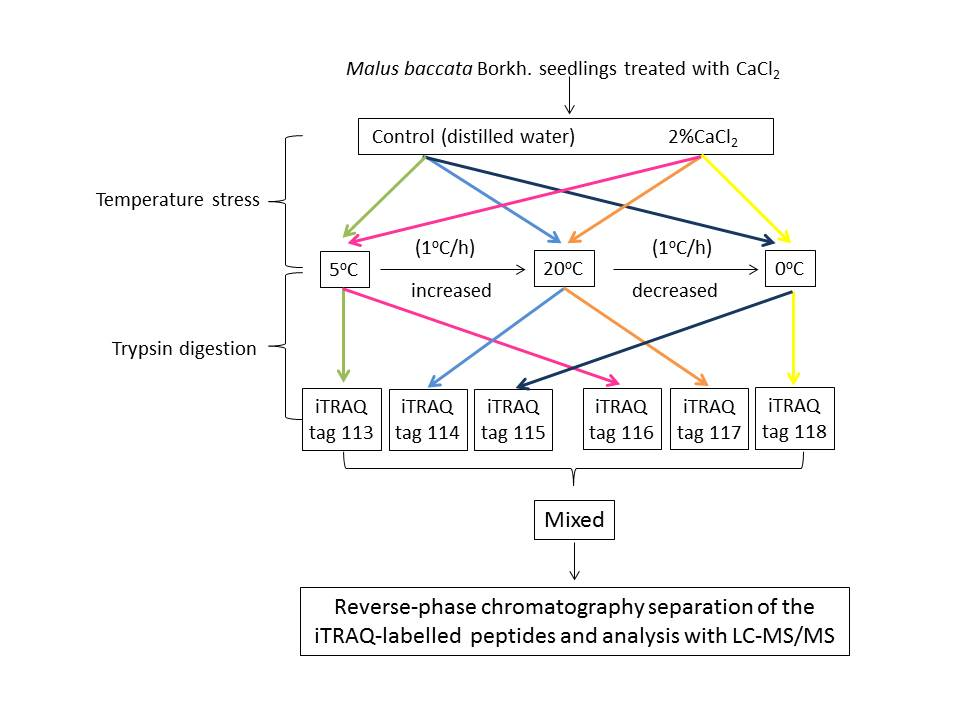

Supplement: Supplementary file 1 [file ijms-18-01755-s001.zip › S Figure 1.tif]
